# Follicular Dendritic Cell Sarcoma of the Spleen: A Report of a Rare Case

**DOI:** 10.7759/cureus.80382

**Published:** 2025-03-11

**Authors:** Rui Jorge Silva, Joana Rita Lopes, Isabel Silva, Sofia Caridade

**Affiliations:** 1 Internal Medicine, Unidade Local de Saúde de Braga, Braga, PRT; 2 Clinical Sciences, School of Medicine, University of Minho, Braga, PRT

**Keywords:** anemia, dendritic cell neoplasm, follicular dendritic cell sarcoma, immune thrombocytopenic purpura, spleen neoplasm, thrombocytopenia

## Abstract

Follicular dendritic cell sarcoma (FDCS) is a rare neoplasm that is composed of cells with morphological and immunophenotypic features of follicular dendritic cells. A minority of these tumors are found on extranodal sites. We describe the case of a 58-year-old woman referred to the hospital emergency department by her general practitioner due to new-onset bicytopenia and petechial rash. The diagnostic workup showed an iron and folic acid-deficient normocytic normochromic anemia, and she was admitted to hospitalization. Further investigation revealed *Helicobacter pylori* infection, later eradicated, and the patient was started on oral dexamethasone over the suspicion of immune thrombocytopenic purpura. An abdominal CT scan was performed that revealed a non-contrast-enhancing 55 mm heterogeneous nodule of the spleen. A PET-CT scan with fluorodeoxyglucose (18F) was also performed, and it revealed a hypermetabolic spleen lesion that raised suspicion of a malignant neoplasm with high metabolic activity. A multidisciplinary team discussed the clinical case, and splenectomy was proposed. After appropriate vaccination, splenectomy was performed, and through macroscopical, microscopical, and immunohistochemical evaluation, the diagnosis of FDCS of the spleen was made. After the splenectomy, the patient presented with bicytopenia resolution, and at the 12-month follow-up, there was no evidence of recurrence of the neoplasm. This clinical case aims to raise awareness about this rare disease and the need for further studies to better understand this entity and its clinical evolution and to define a standard therapeutic protocol, which is currently nonexistent.

## Introduction

Dendritic cell neoplasms encompass several subtypes, such as follicular dendritic cell sarcoma (FDCS) [1]. Follicular dendritic cells (FDCs) are mesenchymal-derived cells located in the B follicles, where they capture, retain, and present antigens to surrounding B cells, playing a critical role in the antigen-dependent humoral immune response phase, which leads to antibody production [1]. First described in 1986, FDCS is a rare neoplasm of lymphoid tissues, characterized by cells with morphological and immunophenotypic features of FDC [1]. It constitutes less than 0.4% of soft tissue sarcomas [2]. FDCS typically manifests within lymph nodes, and in fewer than one-third of cases can also be found in extranodal sites, such as the liver and spleen, with the latter occurrence being described as very rare [3-4]. However, it remains an intriguing tumor, as it may emulate various other tumors or even inflammatory processes [1].

Several primary tumors of the spleen have been described, but the rarity of splenic FDCS poses substantial diagnostic challenges due to their infrequent occurrence and varied clinical features, which may lead to misidentification.

Despite various primary splenic tumors being described, splenic FDCS remains exceptionally rare, presenting substantial diagnostic challenges. This case report explores a rare instance of a splenic dendritic cell tumor, detailing its clinical presentation, diagnostic challenges, and multidisciplinary management.

## Case presentation

A 58-year-old woman was referred to the hospital emergency department by her general practitioner due to new-onset bicytopenia. She had a history of obesity, fibromyalgia, esophagitis, and breast cancer nine years earlier, for which she was treated with chemotherapy with doxorubicin and cyclophosphamide, anti-human epidermal growth factor receptor 2 (HER2) therapy with trastuzumab, and was later subjected to tumorectomy and radiotherapy. She later started hormonotherapy with daily anastrozole, which she maintained until the admission date.

About one week prior to hospital admission, she noticed a petechial rash that started on her lower limbs and was later present on her upper limbs, torso, and face. She also mentioned a self-limited epistaxis episode. Blood work revealed new-onset hemoglobin (Hb) of 9 g/dL (reference range: 11.9-15.6 g/dL) and platelets <10.000/μL (reference range: 150.000-400.000 g/dL), and a referral to the emergency department was made.

In the emergency department, the patient also complained of asthenia and dark-colored feces. She had no other symptoms, including anorexia, weight loss, fever, adenomegaly, night sweats, or abnormal uterine hemorrhage. Her physical examination, apart from discolored mucosae and a petechial rash on her lower limbs, was otherwise unremarkable. She had no macroglossia or hemorrhagic stigma on the oropharynx, no palpable adenomegalies, no palpable breast nodules, no visible hematoma, and no evidence of hemorrhage upon fecal inspection. Her blood work (Table 1) revealed normocytic normochromic anemia, thrombocytopenia, no hemolysis evidence, iron deficit, and folic acid deficit.

**Table 1:** Blood work of the patient in the emergency department

Lab	Value	Unit	Reference range
Hemoglobin	9.1	g/dL	11.9-15.6
Platelets	<10.000	/μL	150.000-400.000
Creatinine	0.8	g/dL	0.6-1.10
Urea	50	g/dL	19-49
Total bilirubin	0.26	mg/dL	0.3-1.2
Direct bilirubin	<0.10	mg/dL	<0.3
Haptoglobin	138	mg/dL	40-280
Aspartate aminotransferase (AST)	32	U/L	12-40
Alanine aminotransferase (ALT)	37	U/L	7-40
Lactate dehydrogenase (LDH)	154	UI/L	120-246
Iron	48	μg/dL	50-170
Ferritin	60	ng/mL	30-322
Total iron binding capacity	325	μg/dL	240-425
Transferrin saturation index	15	%	20-45
Folic acid	5.2	ng/mL	>5.38
Vitamin B12	384	ng/L	211-911

She was started on intravenous iron supplementation and oral folic acid supplementation and was then hospitalized in the internal medicine ward.

Further patient investigation revealed normal serum protein electrophoresis, normal serum immunoglobulin values, previous contact with Epstein-Barr virus, cytomegalovirus, and parvovirus B19, negative hepatitis C and human immunodeficiency viruses, vaccination status for the hepatitis B virus, negative antinuclear antibodies, negative antineutrophil cytoplasmic antibodies, negative lupus anticoagulant, negative beta-2-glycoprotein, and cardiolipin antibodies. The carcinoembryonic antigen (CEA) and carbohydrate antigen 19.9 (CA 19.9) were also negative. The patient’s feces were tested for the presence of *Helicobacter pylori* antigen, which came back positive, so she was started on an eradication regimen composed of pantoprazole, metronidazole, amoxicillin, and clarithromycin. Because the presentation was compatible with immune thrombocytopenic purpura, the patient was started on oral daily 40 mg dexamethasone for four days.

**Figure 1:**
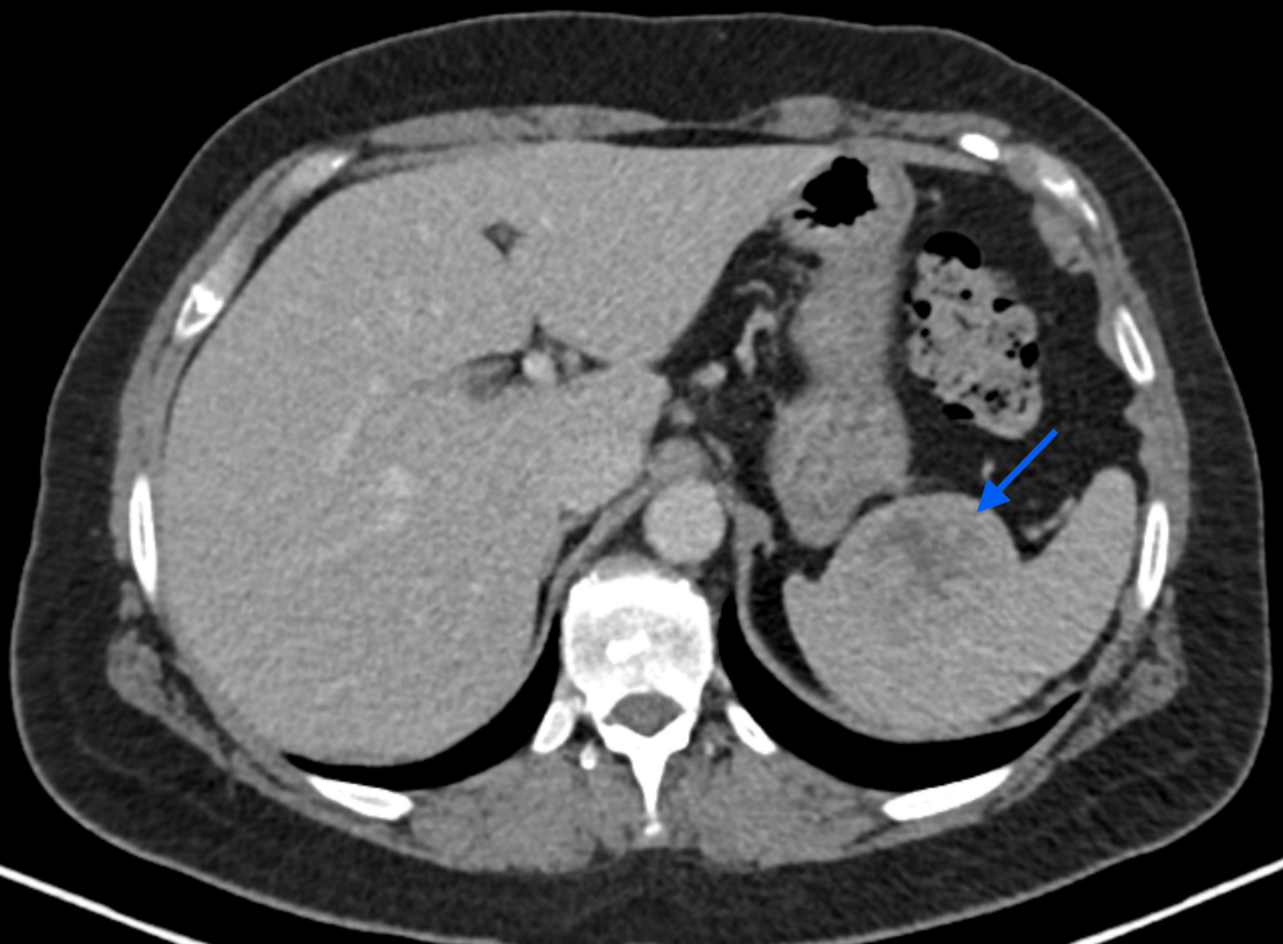
Patient's abdominal axial CT scan Non-contrast-enhancing 55 mm heterogeneous nodule in the spleen signaled with a blue arrow.

On day 9 of hospitalization, the patient performed a contrast-enhanced thoracic, abdominal, and pelvic computerized tomography (CT) scan, which revealed a spleen with normal dimensions but with a non-contrast-enhancing 55 mm heterogeneous nodule (Figure 1).

**Figure 2:**
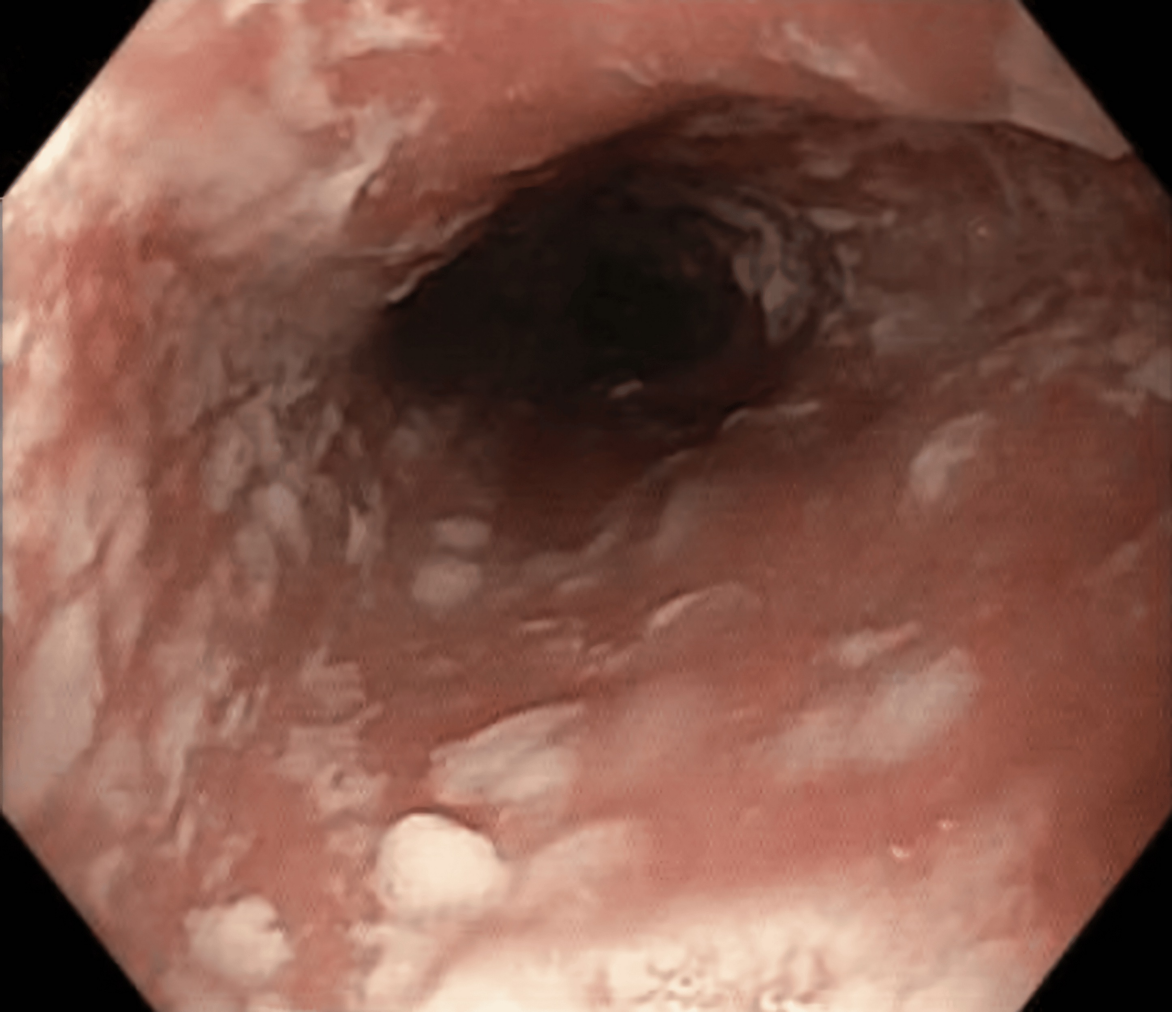
Upper endoscopy showing esophageal candidiasis

Upper and lower endoscopies were also performed, which revealed esophageal candidiasis (Figure 2), so fluconazole treatment was initiated.

Blood analyses were repeated on day 14 of hospitalization, which revealed a Hb of 9.1 g/dL (reference range: 11.9-15.6 g/dL), platelets of 89.000/μL (reference range: 150.000-400.000 g/dL) and adequate medullar response (reticulocytes 5.4% (reference range: 0.5-2.0 g/dL) and reticulocyte proliferation index of 2.4%). To further investigate the radiological findings of the CT scan, a positron emission tomography (PET)-CT scan with fluorodeoxyglucose (18F) was performed. It revealed a hypermetabolic spleen lesion that raised suspicion of a malignant neoplasm with high metabolic activity, with no other suspicious findings.

With all the findings, the clinical case was then discussed in a multidisciplinary team meeting comprised of internal medicine, oncology, and general surgery specialists, and a splenectomy was proposed to the patient. She was prescribed a vaccination regimen for capsulated bacteria (comprised of the vaccines *Haemophilus influenzae* B, *Neisseria meningitidis* B, *Neisseria meningitidis* ACYW135, and *Streptococcus pneumoniae* with 13 serogroups) and for the influenza virus. Upon discharge, the platelet values dropped again to 49.000/μL (reference range: 150.000-400.000 g/dL), so she was started again on oral daily 40 mg dexamethasone for four days, and follow-up consultations were scheduled.

**Figure 3:**
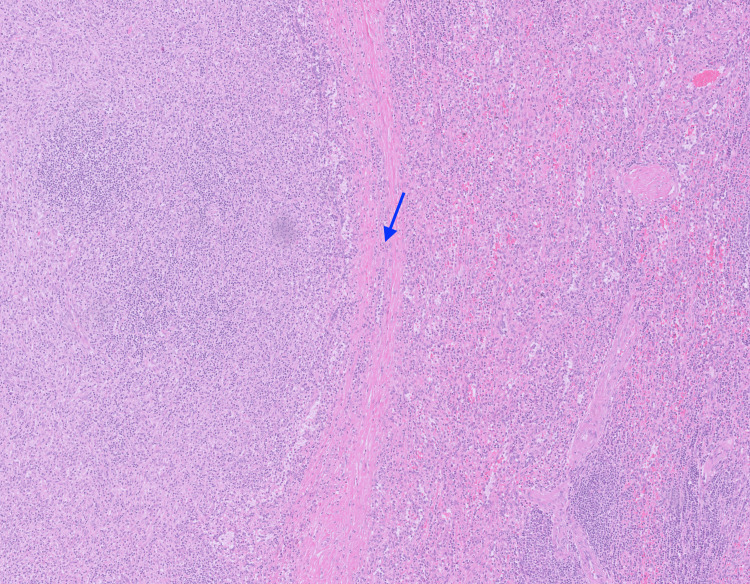
Microscopical examination of the spleen: transition between the normal spleen tissue and the lesion The transition between the normal tissue (right) and the lesion (left) is signaled by a blue arrow.

**Figure 4:**
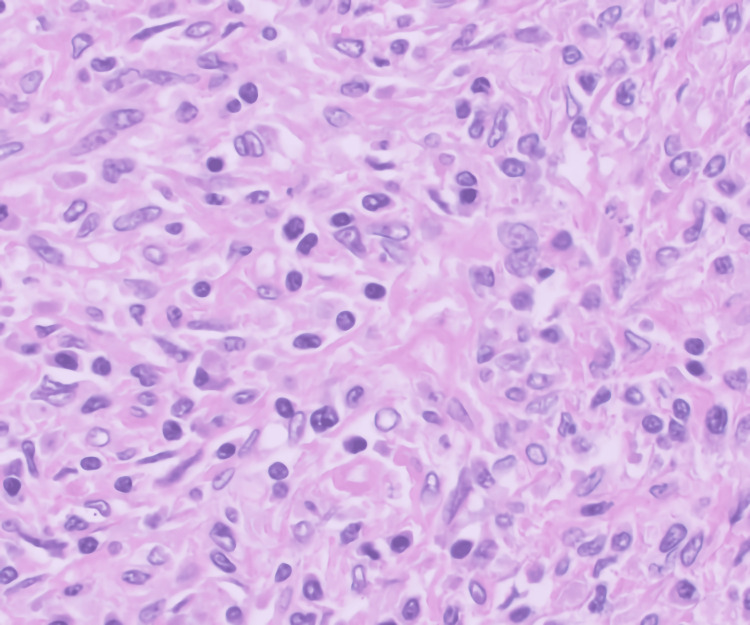
Microscopical examination of the spleen lesion

The patient performed a mammogram and breast ultrasound during the senology consultation, which came back normal. She then performed a third four-day dexamethasone pulse because of falling platelet values, reaching the value of 177.000/μL (reference range: 150.000-400.000 g/dL). Seven weeks after initial hospitalization, the patient was subjected to laparoscopic splenectomy, with no complications noted. Macroscopical examination of the spleen revealed a compact, well-defined nodule with 3.8 x 4 x 2.7 cm dimensions. Microscopical examination revealed a neoplasm predominantly composed of fusiform histiocytic cells with no necrosis or mitosis. We can observe the histology with more details in Figures 3-4.

Immunohistochemical evaluation with the CD10, CD20, CD3, CD4, bcl2, CD5, CD30, CD21, CD23, GATA3, BerEB4, CG79a, and CD8m markers favored the diagnosis of FDCS of the spleen. CD21 and CD23 are FDC markers, which were positive in this case.

After splenectomy, the patient presented with bicytopenia resolution, and at 12 months follow-up, there is no evidence of recurrence of the neoplasm.

## Discussion

FDCS affects both genders with no predilection and typically occurs during adulthood [5]. Patients with FDCS may present a wide range of clinical features. They are typically asymptomatic but may refer to pain due to the slow-growing mass [1]. Other associated conditions have been described, such as paraneoplastic pemphigus (associated or not with myasthenia gravis) and schizophrenia, but FDCS isn't typically associated with lymphomas or leukemias [1]. In this clinical case, the patient was a 58-year-old woman with clinical features of immune thrombocytopenic purpura, namely the petechial rash, which motivated her to seek medical care and the subsequent investigation.

*H. pylori* antigen presence in the feces was tested as the infection by this bacteria has been associated with hematological diseases such as iron deficiency anemia and immune thrombocytopenia [6]. This was also the rationale behind the eradication treatment that was prescribed. However, the complaints of dark-colored feces associated with iron and folic acid-deficient normocytic normochromic anemia made the endoscopies mandatory.

Regarding the immune thrombocytopenic purpura, because the patient had a good hematological response with dexamethasone therapy associated with *H. pylori* eradication and intravenous iron supplementation, the team opted to postpone the medullary study to a later date if it was deemed critical to the diagnosis. The resolution of bicytopenia after splenectomy suggests a causal relationship between the tumor and the purpura (possibly through immune-mediated mechanisms), but we did not find any bibliography supporting this causal relationship.

The radiological exams played a critical role in detecting the spleen mass. Sometimes the characteristics of the mass on the CT scan may point to a particular diagnosis such as cysts, lymphomas, angiosarcomas, or metastases, but this wasn't the case for this patient. Due to the lack of typical findings, the multidisciplinary team proposed splenectomy to the patient, as it would allow for a histologic diagnosis and, as was later revealed, provide treatment.

Regarding the tumor, excisional biopsy remains the preferred method for diagnosing dendritic cell neoplasms, as morphology and immunohistochemical evaluation play a pivotal role in this process and allow distinction from other entities [1]. For localized nodules, such as this patient’s, complete surgical resection is preferred as it presents as an effective treatment and is associated with a favorable prognosis [1,4]. Histological features associated with a worse prognosis include size (≥ 6 cm), necrosis, high mitotic count (≥ 5 mitoses per 10 high-power fields), and significant cytological atypia [1]. However, disseminated disease usually carries a poor prognosis, and there is no standard therapeutic protocol (different approaches may include surgery, radiotherapy, chemotherapy, and tyrosine-kinase inhibitors) [1].

Clinical evolution is characterized by local recurrences in 28% of the cases and distant metastasis (with the main sites being lung, liver, lymph nodes, and bones) in 27% [1]. In this case, local recurrence is impossible since she was subjected to splenectomy, and, as of this article’s writing, the patient has no evidence of distant metastasis. Still, since more research is needed to better understand FDCS, follow-up is mandatory and is being maintained.

## Conclusions

This case highlights a rare instance of FDCS of the spleen, emphasizing its relevance in the differential diagnosis of splenic tumors. Due to its nonspecific clinical presentation and the limitations of noninvasive testing, FDCS remains a diagnostic challenge, often requiring histopathological confirmation following splenectomy. As surgical resection remains the main treatment for localized disease, splenectomy also proved to have a therapeutic role.

Further research is needed to better understand this entity, establish standardized treatment protocols, identify prognostic markers, and determine the role of adjuvant therapies in FDCS management, for optimal patient outcomes.
